# Congenital nystagmus, disability, visual impairment, and noncompaction suggest hereditary disease

**DOI:** 10.31744/einstein_journal/2019CE5251

**Published:** 2019-11-13

**Authors:** Josef Finsterer

**Affiliations:** 1 Neurological Department, Krankenanstalt Rudolfstiftung, Messerli Institute, Vienna, Austria.

Concerning the article by Maia et al.,^1^we have the following comments. Since noncompaction also known as left ventricular hypertrabeculation (LVHT) is frequently associated with genetic disease,^2^ first-degree family members should be screened for LVHT, visual impairment, and nystagmus. Disability and visual impairment should be specified and results of cerebral magnetic resonance imaging presented.

Left ventricular hypertrabeculation is not congenital in each case. Acquired LVHT has been reported, particularly in patients with neuromuscular disorders (NMDs), professional athletes, and pregnant women.^3^ Previous echocardiographies should be revised. If LVHT was acquired in the patient of the study, a NMD should be considered since the patient was not pregnant nor a professional athlete.

It should be discussed why LVHT was not detected on echocardiography but only on cardiac magnetic resonance imaging. According to which criteria was LVHT diagnosed on cardiac magnetic resonance imaging? Left ventricular hypertrabeculation is frequently associated with late gadolinium enhancement.^4^ Was late gadolinium enhancement present on cardiac magnetic resonance imaging?

Overall, the patient could profit from addressing the points mentioned above.
