# Supplementary material for: Congenital nystagmus, disability, visual impairment, and noncompaction suggest hereditary disease
Source: Einstein (Sao Paulo). 2019 Nov 13;17(4):eCE5251. doi: 10.31744/einstein_journal/2019CE5251 (PMC6855696; doi:10.31744/einstein_journal/2019CE5251)
Supplement: Translation

# Translation: Nistagmo congênito, deficiência, disfunção visual e não compactação sugerem doença hereditária

**Authors:** Josef Finsterer

**Affiliations:** 1 Departamento de Neurologia, Krankenanstalt Rudolfstiftung, Messerli Institute, Viena, Áustria.

Em relação ao artigo de autoria de Maia et al.,^( 1 )^faço os comentários a seguir. Considerando que a não compactação do ventrículo esquerdo (NCVE) é frequentemente associada com doença genética,^( 2 )^membros da família de primeiro grau devem ser examinados para NCVE, disfunção visual e nistagmo. A deficiência e a disfunção visual devem ser específicas, e os resultados do exame de imagem por ressonância magnética cerebral devem ser apresentados.

A NCVE não é congênita em cada caso. A NCVE adquirida tem sido relatada, principalmente em paciente com distúrbios neuromusculares (DNMs), atletas profissionais e gestantes.^( 3 )^As ecocardiografias anteriores devem ser revisadas. Se a NCVE foi adquirida pelo paciente do estudo, um DNM deveria ser levado em consideração, já que o paciente não era gestante e nem atleta profissional.

Deve-se discutir o motivo pelo qual a NCVE não foi detectada na ecocardiografia, mas somente por meio da ressonância magnética cardíaca. Qual foi o critério para diagnosticar NCVE por meio do exame de ressonância magnética cardíaca? Geralmente, a NCVE é associada a realce tardio com gadolínio.^( 4 )^Observou-se realce tardio com gadolínio no exame de ressonância magnética?

Possivelmente, o paciente poderia ter se beneficiado se os pontos mencionados fossem abordados.
